# Prediction of intraoperative cerebrospinal fluid leaks in endoscopic endonasal transsphenoidal pituitary surgery based on a deep neural network model trained with MRI images: a pilot study

**DOI:** 10.3389/fnins.2023.1203698

**Published:** 2023-07-27

**Authors:** Hui Chang, Kai Zhao, Jun Qiu, Xiang-Jun Ji, Wu-Gang Chen, Bo-Yuan Li, Cheng Lv, Zi-Cheng Xiong, Sheng-Bo Chen, Xu-Jun Shu

**Affiliations:** ^1^School of Computer and Information Engineering and Henan Engineering Research Center of Intelligent Technology and Application, Henan University, Kaifeng, Henan Province, China; ^2^The First Medical Center, Chinese PLA General Hospital, Beijing, China; ^3^Department of Critical Care Medicine, The Second People’s Hospital of Yibin, Yibin, Sichuan Province, China; ^4^Department of Neurosurgery, Jinling Hospital, Medical School of Nanjing University, Nanjing, Jiangsu Province, China; ^5^School of Mathematics and Computer Sciences, Nanchang University, Nanchang, Jiangxi Province, China

**Keywords:** cerebrospinal fluid leaks, endoscopic endonasal transsphenoidal surgery, pituitary adenoma, deep neural network model, neural network

## Abstract

**Objective:**

This study aimed to investigate the reliability of a deep neural network (DNN) model trained only on contrast-enhanced T1 (T1CE) images for predicting intraoperative cerebrospinal fluid (ioCSF) leaks in endoscopic transsphenoidal surgery (EETS).

**Methods:**

396 pituitary adenoma (PA) cases were reviewed, only primary PAs with Hardy suprasellar Stages A, B, and C were included in this study. The T1CE images of these patients were collected, and sagittal and coronal T1CE slices were selected for training the DNN model. The model performance was evaluated and tested, and its interpretability was explored.

**Results:**

A total of 102 PA cases were enrolled in this study, 51 from the ioCSF leakage group, and 51 from the non-ioCSF leakage group. 306 sagittal and 306 coronal T1CE slices were collected as the original dataset, and data augmentation was applied before model training and testing. In the test dataset, the DNN model provided a single-slice prediction accuracy of 97.29%, a sensitivity of 98.25%, and a specificity of 96.35%. In clinical test, the accuracy of the DNN model in predicting ioCSF leaks in patients reached 84.6%. The feature maps of the model were visualized and the regions of interest for prediction were the tumor roof and suprasellar region.

**Conclusion:**

In this study, the DNN model could predict ioCSF leaks based on preoperative T1CE images, especially in PAs in Hardy Stages A, B, and C. The region of interest in the model prediction-making process is similar to that of humans. DNN models trained with preoperative MRI images may provide a novel tool for predicting ioCSF leak risk for PA patients.

## 1. Introduction

Endoscopic endonasal transsphenoidal surgery (EETS) has been established as the primary treatment for pituitary adenomas (PAs) due to its high rate of gross-total resection (GTR), low postoperative morbidity, and low mortality (Chen et al., 2017; Campana et al., 2022). A major complication of EETS is postoperative cerebrospinal fluid (CSF) leakage, potentially leading to intracranial infection and meningitis (Van Aken et al., 2004; Laws et al., 2013; Conger et al., 2018). After EETS, the incidence of postoperative CSF leakage ranges from 0.5 to 12% (Dehdashti et al., 2008; Paluzzi et al., 2014; Wang et al., 2015). Factors associated with postoperative CSF leakage include surgery for macroadenomas, repeated EETS, and higher body mass index (BMI; Karnezis et al., 2016; Patel et al., 2018; Xue et al., 2020; Li et al., 2022). However, intraoperative CSF (ioCSF) leaks is the most commonly reported predictor of postoperative CSF rhinorrhea (Karnezis et al., 2016; Magro et al., 2016; Hannan et al., 2020). Identifying patients at high risk of ioCSF leakage preoperatively is essential as it may change the closure strategy of EETS, requiring the use of autologous fat and vascularized nasoseptal flap to reconstruct sella turcica (Esposito et al., 2007; Koutourousiou et al., 2013; Wang et al., 2015; Conger et al., 2018).

It has been reported that a diaphragmatic defect resulting from suprasellar invasion of the PA is responsible for ioCSF leaks (Conger et al., 2018; Li et al., 2022). According to the Hardy-Wilson modified scale, tumor extrasellar extension is divided into five Stages: Stage 0, with no suprasellar extension. Stage A, tumor occupying suprasellar cistern. Stage B, recess of third ventricle obliterated. Stage C, third ventricle displaced. Stage D, intracranial extension. Stage E, intracranial extension and cavernous sinus extension. PAs in Stage 0 are associated with a low risk of diaphragmatic defect, while those in Stages D and E indicate a higher risk of diaphragmatic defect (Li et al., 2022). Previous studies using multivariate logistic regression showed that large tumors, irregular tumor contours, and tumor texture consistencies are prominent risk factors for ioCSF leaks (Karnezis et al., 2016; Patel et al., 2018; Xue et al., 2020; Li et al., 2022). These risk factors, however, cannot be quantified and used to predict ioCSF leaks in PA patients, especially those with Hardy suprasellar Stages A, B, and C.

Artificial intelligence and deep learning have revolutionized the fields of medicine and neurosurgery. Deep neural network (DNN) models can now predict various clinical tasks that would have been impossible using previous statistical models. Magnetic resonance imaging (MRI) is a non-invasive imaging technology that is used for the diagnosis and treatment of PAs routinely. MRI contains the information of tumor size, contour, and consistency of PAs, thus making it the ideal data source for DNN model training.

In this study, we investigated whether a DNN trained using only contrast-enhanced T1 (T1CE) images could predict ioCSF leakage. Additionally, the DNN model was tested in clinical settings, and model interpretability was also explored.

## 2. Methods

### 2.1. Patients

We reviewed 396 PA patients who underwent EETS between May 2017 and October 2022 at the Department of Neurosurgery, Jinling Hospital, retrospectively. The preoperative and postoperative MRI images and surgical records were routinely collected, and all patients were treated according to the same surgical protocol by two senior surgeons. The ethical committee of Jinling Hospital approved the study registry and data collection.

**Figure 1:**
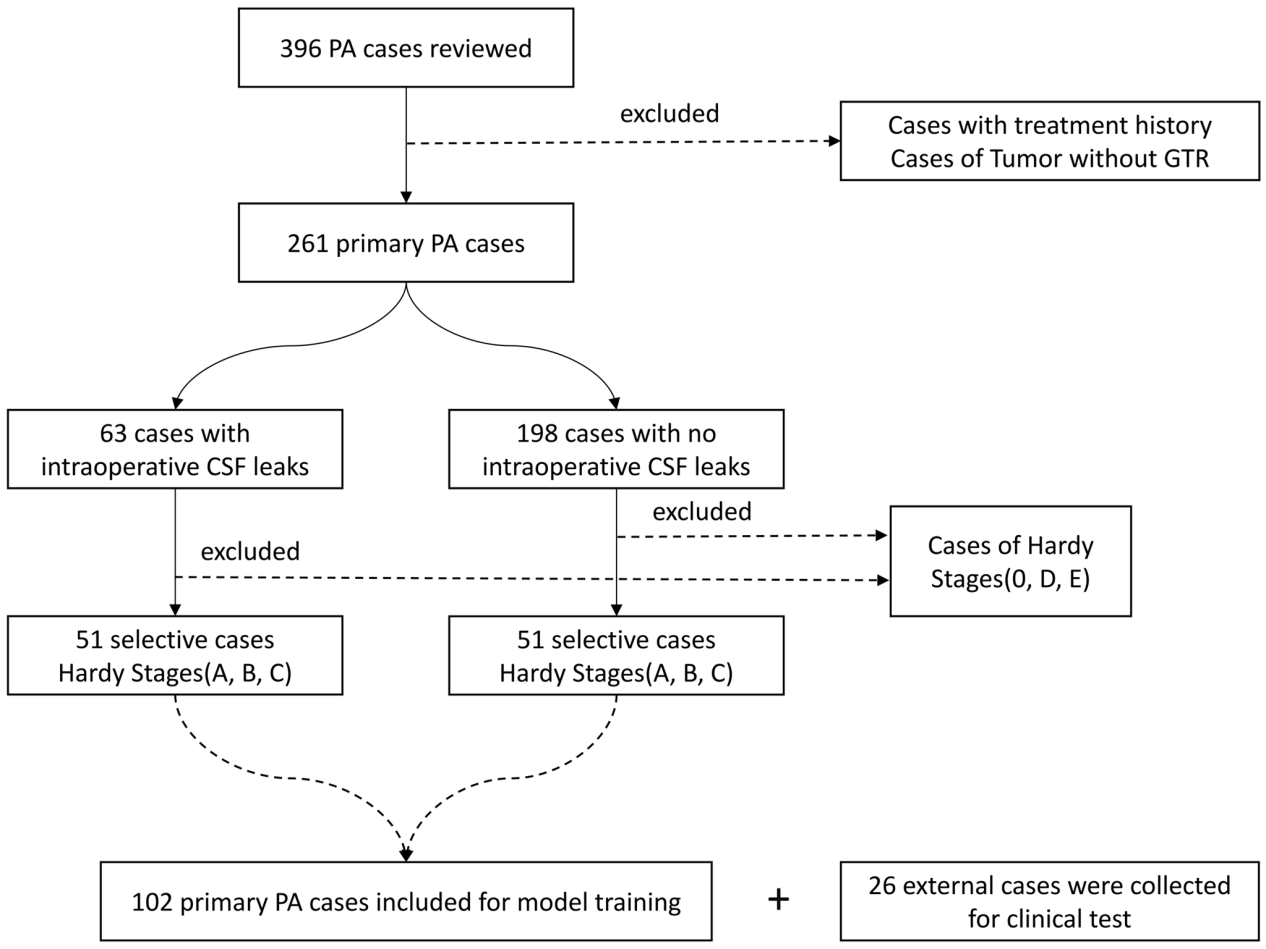
Flowchart of patient selection. PA, pituitary adenoma; GTR, gross total resection; and CSF, cerebrospinal fluid.

We excluded cases of PAs in Hardy suprasellar Stages 0, D, and E, as well as cases of PAs without gross-total-resection. Additionally, recurrent cases and cases with a history of medical therapy were also excluded to make sure that diaphragmatic defects were caused by tumors. Consequently, a total of 51 cases were included in the ioCSF leakage group. To investigate model interpretability, we randomly selected 51 cases with primary PAs in Hardy suprasellar Stages A, B, and C from the non-intraoperative CSF (non-ioCSF) leakage group to balance the control group. Finally, a total of 102 cases were enrolled in this study. In the clinical test, an external dataset from 26 consecutive cases with primary PA in Hardy Stages A, B, and C was tested (Figure 1).

### 2.2. MRI images collection

MRI images of all patients were acquired before surgery using a 1.5 Tesla scanner (Siemens Espree, Erlangen, Germany). DICOM images of axial T1CE with a thickness of 1 mm were collected. The parameters for T1CE were as follows, Slice thickness = 1 mm, Field-of-view = 130 mm, Matrix size = 512 × 512 × 176, Flip angle = 15^°^, Echo time = 3.02 ms, Repetition time = 1,650 ms, and Voxel dimensions = 0.997 × 0.997 × 1 mm^3^. Coronal and sagittal T1CE images were reconstructed from thin-sliced axial T1CE images with 3D Slicer software (version 4.10, Harvard University, Boston, United States).

### 2.3. Tumor segmentation

IWS software (Version 1.0, Medinsightech Corp., Shanghai, China) were used for sorting and de-identification of DICOM images. DICOM images of T1CE were then converted into NIFTI images using MRI Convert software (Version 2.1, University of Oregon, Eugene, United States). T1CE NIFTI images were manually segmented by a senior radiologist (Xiang-jun Ji) using ITK-Snap software (Version 3.8, University of Pennsylvania, Philadelphia, United States). Segmentation labels for PAs were saved as tumor masks.

### 2.4. Image preprocessing

#### 2.4.1. Re-sample and normalization

Axially isotropical re-sampling of MRI images to 1 mm × 1 mm × 1 mm using the B-Spline of order 1 interpolation algorithm. MRI images were normalized to reduce the inconsistency of gray-scale information in identical tissues caused by differences in equipment acquisition and scanning parameters, while still preserving the diagnostic value of gray-scale differences.

#### 2.4.2. Slice selection

T1CE images with PAs were selected based on the tumor mask. For each patient, three coronal and three sagittal slices with the largest tumor size were selected. The labels of the six slices were the same as the patient’s. Finally, 306 coronal and sagittal slices labeled as ioCSF leakage and 306 coronal and sagittal slices labeled as non-ioCSF leakage were obtained as the original dataset for further processing.

#### 2.4.3. Slice resize

Since the coronal and sagittal slices of the MRI had different dimensions, the slices need to be uniformly resized so that we can use coronal and sagittal slices for model training. The size of the sagittal slice was 250*176 pixels, while the coronal slice was 188*176 pixels. In this study, both coronal and sagittal slices were uniformly resized to 192 *192 pixels.

#### 2.4.4. Data augmentation

Data enhancement techniques such as exponential and logarithmic transforms, histogram equalization, power transforms, Laplace image sharpening, adding Laplace noise and left–right rotation were used. After data augmentation, a total of 4,896 slices were obtained, half with ioCSF leakage labels and the other half with non-ioCSF leakage labels.

### 2.5. Neural network architecture

**Figure 2:**
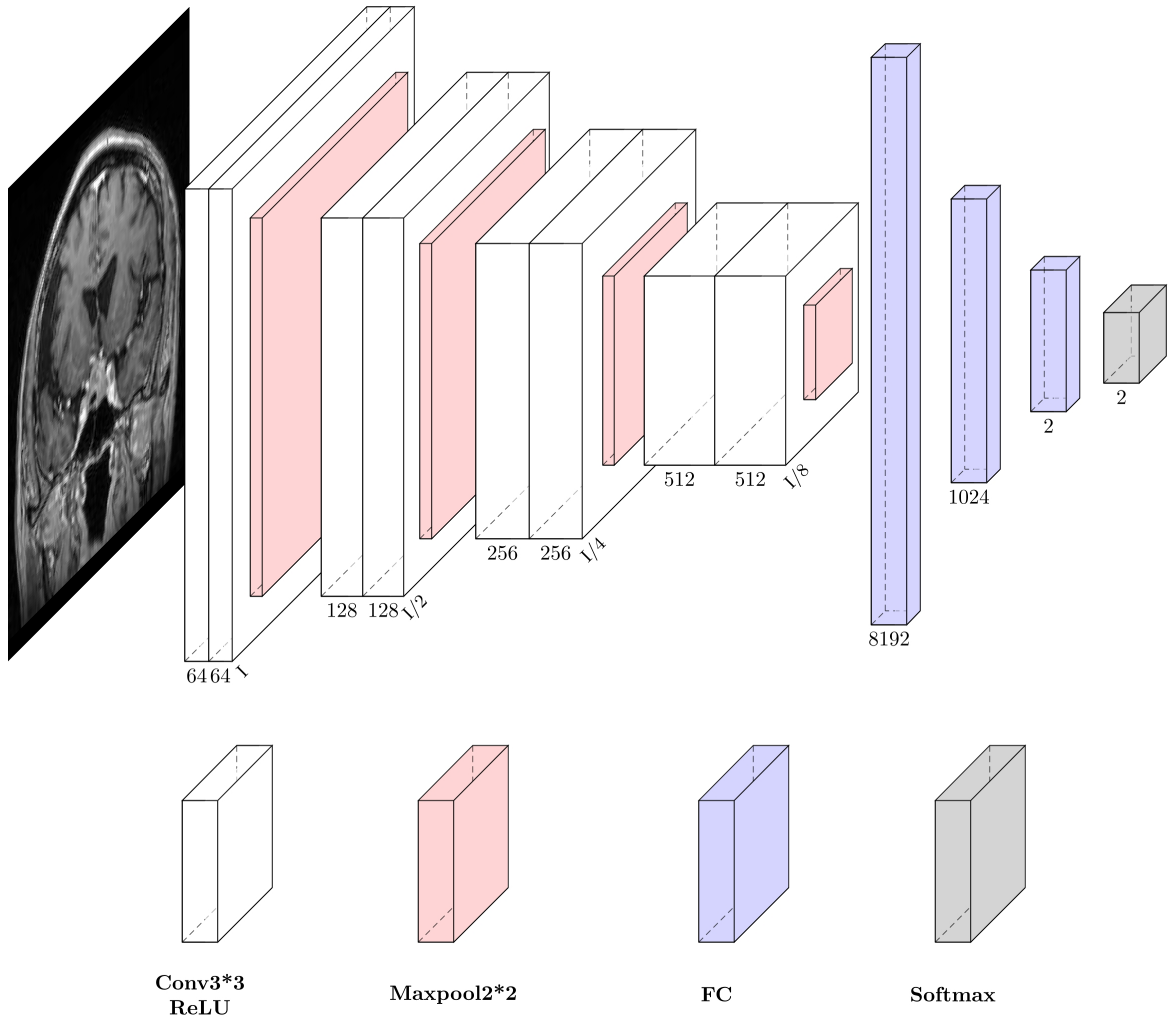
Neural network architecture. Each down-sampling module consists of two 3*3 convolution layers and a 2*2 max pooling layer, which is used for image down-sampling to extract features. After three fully connected layers, the final softmax is used as the estimation of the final class distribution. Conv, convolution; ReLU, rectifiers linear unit; FC, full connection layer.

U-Net is one of the most commonly used neural networks for biomedical image segmentation. For our prediction task, we utilized a customized U-Net architecture. Only the down-sampling part of the U-net was kept to extract features, followed by several fully connected layers and dropout layers for conclusive classification. The neural network architecture is shown in Figure 2.

The size of the input image is 192*192 pixels. The down-sampling process consisted of four modules. Each module contained two convolution operations and one max pooling operation. The feature map decreased in size and the perceptual field grew larger as the depth of the neural network increased, while extracting high-level features. The last feature map passes through a sequence of three fully connected layers and the final output is produced via the softmax layer. To reduce overfitting, two dropout layers were integrated into the fully connected layers. Throughout the training process, the feature maps provided comprehensible visualization.

### 2.6. Model training and validation

Before model training, 400 slices from each group were randomly selected as the testing dataset. The remaining 4,096 slices were randomly shuffled and divided into 80% for model training and 20% for model validation. The hyperparameters in the model training were set as follows: using SGD as the optimizer, cross-entropy as the loss function, a learning rate of 0.0001, a batch size of 128, a momentum of 0.9, and a weight decay of 0.0001. The learning rate decreased by 5% every five epochs. The evaluation metrics of model performance included accuracy, sensitivity, and specificity. The formulas are as follows:


$$\text{Accuracy}=\frac{\text{TP}+\text{TN}}{\text{TP}+\text{FN}+\text{TN}+\text{FP}}$$

$$\text{Sensitivity}=\frac{\text{TP}}{\text{TP}+\text{FN}}$$

$$\text{Specificity}=\frac{\text{TN}}{\text{TN}+\text{FP}}$$

where TP is the true positive, TN is the true negative, FN is the false negative and FP is the false positive. 5-fold cross-validation was also performed on the training dataset to evaluate the model performance.

### 2.7. DNN model workflow

**Figure 3:**
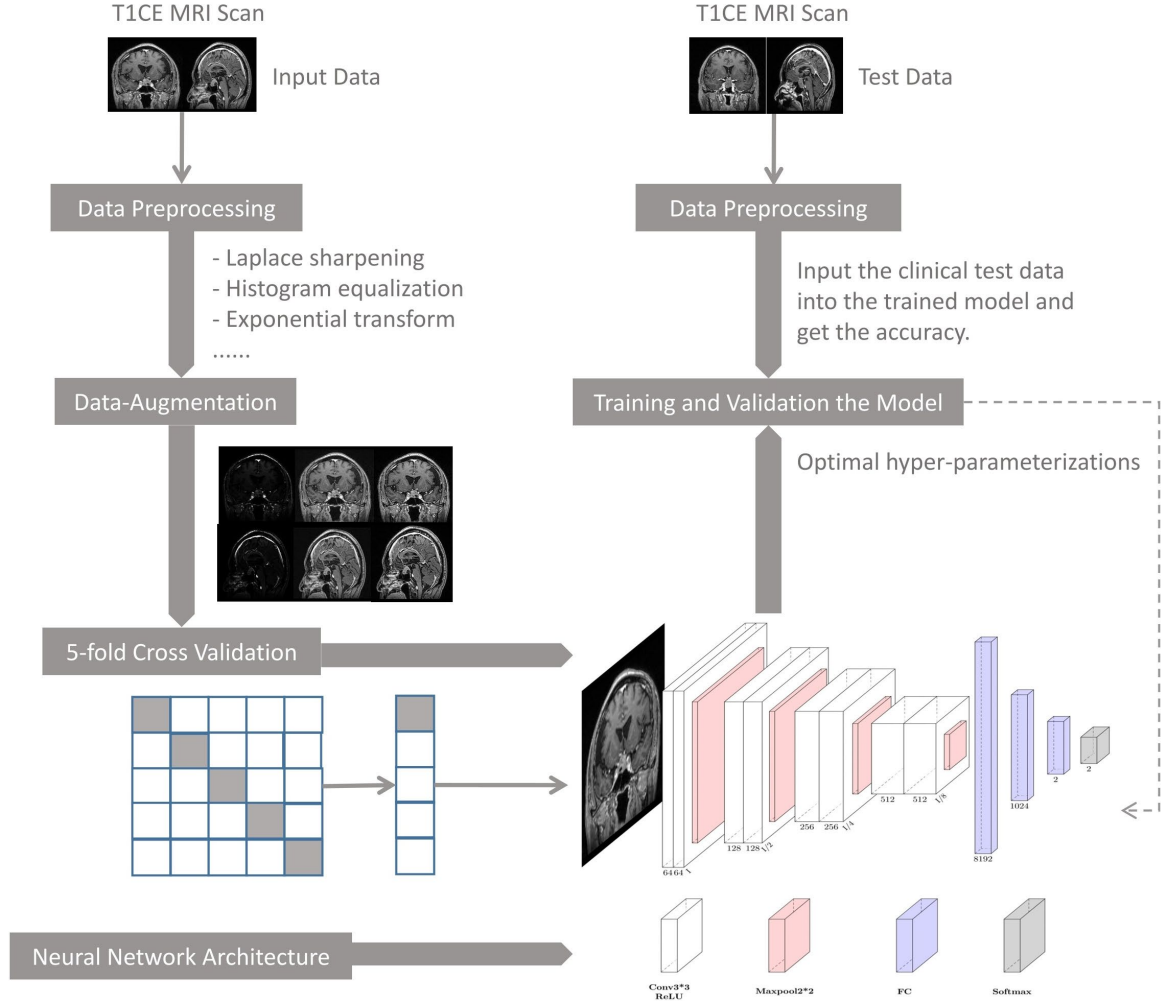
Flowchart of deep learning development.

The flowchart of the DNN model is depicted in Figure 3, where its main components are: (1) data preprocessing, (2) model training and validation, (3) model performance evaluation, and (4) clinical testing.

### 2.8. Clinical test and human-machine comparison

Our DNN model makes the prediction based on the single slice. When used to make predictions on patients, different slices from one patient may produce conflicting predictions by the DNN model. To address this problem, we introduced a slice-voting strategy that converted slice-based predictions into patient-based predictions. Three coronal and three sagittal slices containing the maximum tumor size were selected from a single patient. Data augmentation was also used to increase the number from 6 to 48 slices. Forty-eight results from 48 slices voted for the final prediction of the patient.

In clinical test, two senior experts with more than 10 years of experience in pituitary surgery predicted the occurrence of ioCSF leaks for each PA patient before surgery. The prediction accuracy of the two experts was then compared to that of the DNN model.

### 2.9. Statistical analysis

All statistical analysis were performed using SPSS 19.0 (IBM Corp., Armonk, New York, United States). A *t*-test was performed to determine the difference between two groups in Hardy’s suprasellar Stages A, B, and C. *p* value <0.05 was considered statistically significant.

## 3. Results

### 3.1. Patient information and PA characteristics

A total of 102 patients with primary PAs in Hardy suprasellar Stages A, B, and C were included in the study. In the ioCSF leakage group, there were 27 female and 24 male patients. In the non-ioCSF leakage group, there were 24 female and 27 male patients. The average age for the ioCSF leakage group and the non-ioCSF leakage group were 49.0 and 49.5 years old, respectively. The average value of tumor maximum diameters in the ioCSF leakage and non-ioCSF leakage groups were 24.6 and 20.9 mm, respectively. The average volume of tumors in the ioCSF leakage and non-ioCSF leakage groups were 3.9 and 3.5 cm^3^, respectively. The ioCSF leakage group had 27 nonfunctional PAs, while the non-ioCSF leakage group had 25 nonfunctional PAs. In the ioCSF leakage group, there were 12 PAs in the Stage A, 15 PAs in the Stage B, and 24 PAs in the Stage C. In the non-ioCSF leakage group, there were 10 PAs in Stage A, 19 PAs in the Stage B, and 21 PAs in the Stage C (Table 1).

**Table 1:** Patient information and PA characteristics.

	Non-ioCSF leakage	ioCSF leakage	*p* value
Female	27(52.9%)	21(41.2%)	
Age (years)	49.5 ± 13.9	49.0 ± 14.9	0.860
PA max diameter (mm)	20.98 ± 8.8	24.63 ± 8.3	0.245
PA volume (cm^3^)	3.495 ± 2.8	3.898 ± 3.5	0.289
Nonfunctional PA	25(49.0%)	27(52.9%)	0.741
Hardy stage (suprasella)			
A	10(19.6%)	12(23.5%)	
B	19(37.3%)	15(29.4%)	
C	21(41.2%)	24(47.1%)	

PA, pituitary adenoma; ioCSF, intraoperative cerebrospinal fluid; yrs, years; mm, millimeter.

### 3.2. Model training, validation, and testing

**Figure 4:**
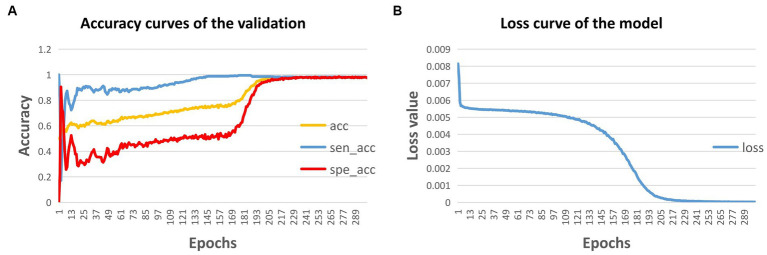
Accuracy curve **(A)** and loss curve **(B)** of the model during training. acc, accuracy; sen, sensitivity; and spe, specificity.

In this study, the model was trained for 300 epochs, and the trends of the accuracy of the validation dataset and the training loss are illustrated in Figure 4. Based on the 5-fold cross-validation, the average accuracy on the validation dataset was 97.62%, while the average sensitivity and specificity were 98.31 and 96.92%, respectively. Moreover, on the test dataset, the prediction accuracy was 97.29%, with a sensitivity of 98.25% and a specificity of 96.35%. (Table 2).

**Table 2:** Model performance evaluated on five-fold cross-validation and on testing dataset.

	Validation dataset						Testing dataset
	The first fold	The second fold	The third fold	The fourth fold	The fifth fold	Average	
Acc	97.89%	97.02%	97.27%	97.64%	98.28%	97.62%	97.29%
Sen_Acc	98.76%	99.00%	98.26%	97.52%	98.03%	98.31%	98.25%
Spe_Acc	97.02%	95.04%	96.28%	97.77%	98.52%	96.92%	96.35%

Acc, accuracy; Sen, sensitive; Spe, specificity.

### 3.3. Clinical test and human-machine comparison test

In the clinical test, an external 26 patients with primary PAs in Hardy suprasellar Stages A, B, and C were tested consecutively. Four out of 26 patients observed ioCSF leaks in surgery. The prediction accuracies of Expert 1 and Expert 2 were 53.85% and 42.31%, respectively. The DNN model achieved a prediction accuracy of 84.46%, which is more than 30% higher than that of the two experts (Table 3).

**Table 3:** Human-machine comparison test in the clinical test.

Patient prediction Acc	Expert 1	Expert 2	DNN model
ioCSF leakage cases	50% (2/4)	25% (1/4)	75% (3/4)
non-ioCSF leakage cases	54.55% (12/22)	45.45% (10/22)	86.36% (19/22)
Total	53.85% (14/26)	42.31% (11/26)	84.61% (22/26)

Acc, accuracy; ioCSF, intraoperative cerebrospinal fluid; DNN, deep neural network.

### 3.4. Interpretability of the DNN model

**Figure 5:**
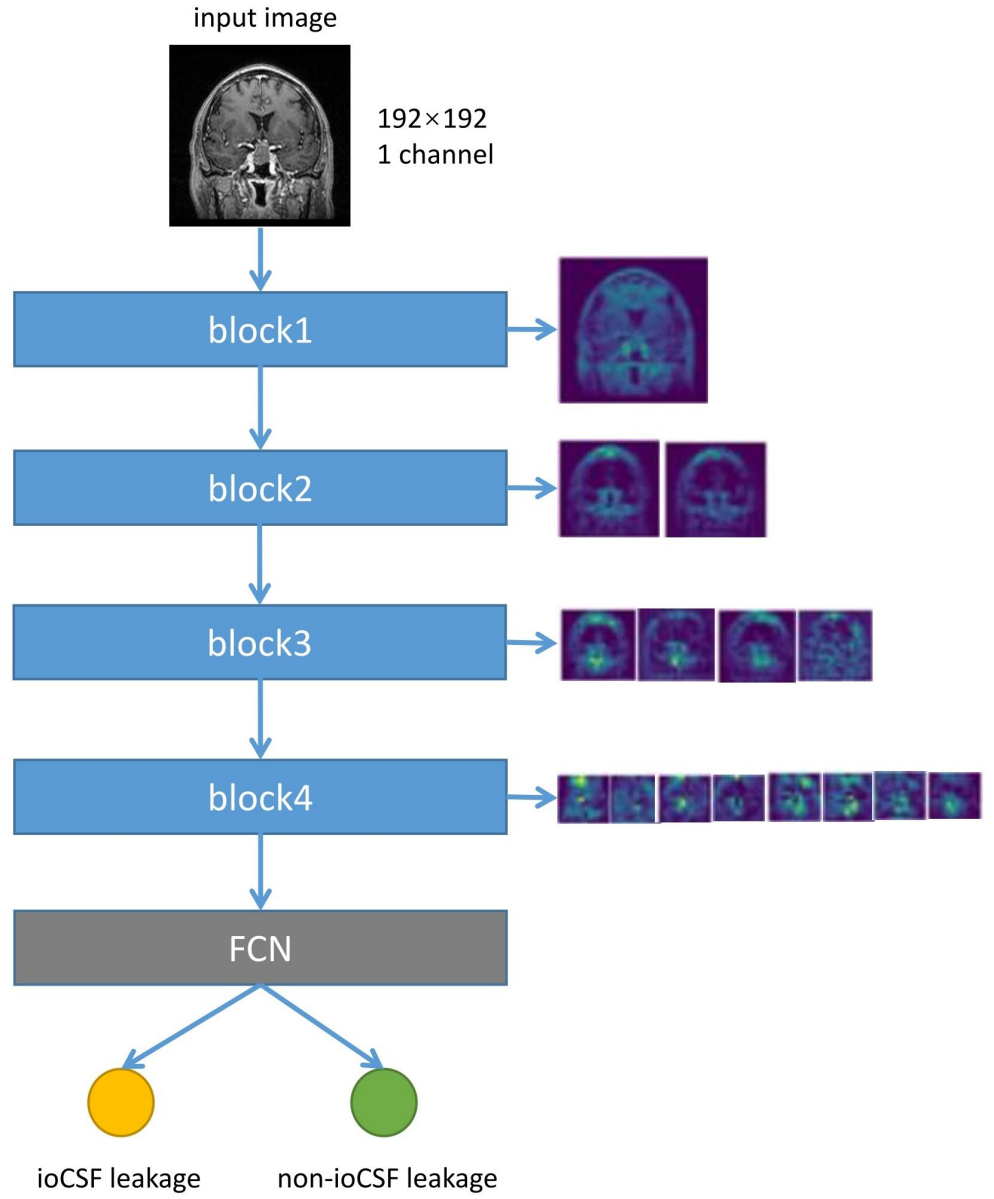
The flow chart of the neural network converting the input image into the feature map, and the visualization of the feature map of different down-sampling modules. The input image size is 192*192 pixels. Each down-sample block halves the size of the image and doubles the number of feature channels. The fully connected layer and softmax layer calculated class scores based on the final feature map to predict ioCSF leaks.

**Figure 6:**
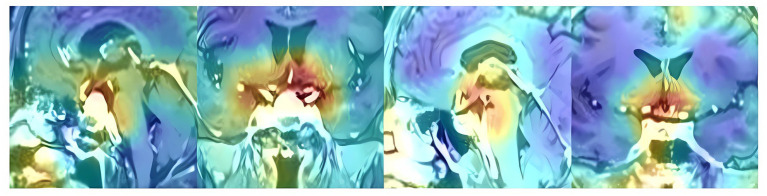
The heatmaps in the sagittal and coronal planes show the hotspot regions that the DNN model focused when making prediction of ioCSF leaks.

To investigate the interpretability of the DNN model, feature map visualization was generated during the whole model training process (Figure 5). Heatmaps were applied to highlight the regions of interest for the DNN model during the prediction-making process. Figure 6 shows that the DNN model focuses on the upper part of the tumor and the suprasellar regions, which contain diaphragmatic information. Similarly, the DNN model focused on the same regions as the experts when predicting the occurrence of ioCSF leaks.

## 4. Discussion

Endoscopic transsphenoidal surgery currently serves as the gold standard for treating PAs. However, a considerable number of patients suffer from postoperative CSF leaks following ioCSF leaks (Mehta and Oldfield, 2012; Karnezis et al., 2016; Mooney et al., 2017). As a key predictor of postoperative CSF rhinorrhea, ioCSF leaks have an incidence rate of 2.8–41% during transsphenoidal procedures (Mehta and Oldfield, 2012; Karnezis et al., 2016; Chen et al., 2017; Przybylowski et al., 2017; Strickland et al., 2017; Patel et al., 2018). In this study, 61 cases out of 261 cases (24.1%) were observed with ioCSF leaks. Significant risk factors for ioCSF leaks in EETS include tumor consistency, tumor size, and repetitive surgery (Karnezis et al., 2016; Xue et al., 2020; Li et al., 2022). To ensure that factors other than the tumor itself, such as radiotherapy, drugs, and repeat surgery, were not involved, only primary PA cases were included in this study. To minimize surgical effects on ioCSF leaks, two senior neurosurgeons with more than 10 years of pituitary surgery experience performed all EETS procedures using the same surgical protocol.

Previous studies that utilized the multivariate logistic regression method revealed that tumor size, texture, irregular upper tumor contour, gonadotrophic-positive staining, and higher BMI in patients were risk factors for ioCSF leaks (Karnezis et al., 2016; Zhou et al., 2017; Xue et al., 2020; Li et al., 2022). However, traditional statistical models cannot quantitatively analyze these risk factors and provide a straightforward prediction when applied to a single PA patient. Machine learning models can achieve much higher prediction performance than logistic regression models. Staartjes et al. (2019) used patients’ sex, age, tumor diameter, tumor volume, Knosp grade, and Hardy grade as input features to train their neural network model, which provided a prediction accuracy of 90% on the validation dataset and 88% on the testing dataset. In a comparison of the model performances of machine learning (Random Forest) and conventional statistical methods (multivariable logistic regression), Mattogno et al. showed that machine learning models had a 30% higher prediction accuracy than conventional statistical models (Qian et al., 2020). In this study, we utilized T1CE images instead of clinical features to train our DNN model. The reasons are as follows: Firstly, T1CE images contain a number of risk factors for ioCSF leaks, including tumor size, shape, and texture. Secondly, T1CE images are more objective than clinical features extracted by clinicians. Finally, in clinical settings, MRI images are more convenient to use as input data for DNN models than models based on multiple features.

In this study, only primary PAs in Hardy Stages A, B, and C were included. The reasons are as follows: first, the prediction of ioCSF leaks for PAs in Hardy Stages A, B and C was challenging, even for experts. Second, PAs in Hardy Stages A, B, and C are the most common types encountered in clinical practice. Finally, limiting the spectrum of PA cases would reduce the long tail effect, simplify model training, and be helpful in investigating the model’s interpretability.

We used a customized U-Net architecture as our neural network. The U-Net is frequently adopted for semantic segmentation in the field of biomedical imaging. Semantic segmentation involves the need for pixel-level discrimination and the transfer of learned features from various encoder stage to each pixel. Unlike semantic segmentation, our objective was to predict the occurrence of ioCSF leaks in EETS for a given PA. We exclude the upsampling component of U-Net because restoring image resolution through upsampling is redundant and may lead to model overfitting.

Three consecutive slices were selected, containing the largest tumor size in both sagittal and coronal views, in order to incorporate more comprehensive information regarding tumor size, texture, and upper contour. Data augmentation can expand the training data and improve the generalization ability of the model. The implementation of data augmentation significantly improved the performance of our DNN model on both validation and testing datasets, with an average accuracy rate of 97.62% on the validation dataset and 97.29% on the testing dataset.

In the clinical test, a slice-voting strategy algorism was used to make the DNN model could make direct predictions on PA patients. Twenty-six patients with primary PAs in Hardy Stages A, B, and C were tested preoperatively. The DNN model gave a prediction accuracy of 84.61%. Compared with the result on the test dataset, it had dropped by 12%, but it was 30% higher than the accuracy predicted by the two experts.

Deep learning is often referred to as a “black box” algorithm because it is not clear to users how the models extract features to make the final predictions. To explore model interpretability, we restricted data enrollment criteria (only PAs in Hardy Stages A, B, and C were included in this study) and performed data balance between the two groups (random selection 51 cases in the control group). Additionally, feature visualization techniques were deployed to observe feature maps from image input to result output, and heatmaps were used to show the regions of interest that the DNN model focused on during prediction. Interestingly, the heatmaps showed that the hotspots were in the tumor roof and suprasellar regions, which coincided with human experts’ focus on predicting ioCSF leaks.

### 4.1. Limitations

Although our DNN model demonstrated excellent performance, the results were based on a small dataset of a single MR Scanner in a single center, which may have limited the reliability of our model in an external patient population with different MR Scanners and different centers. However, this pilot study demonstrates the feasibility of the DNN model to predict ioCSF leaks for PA patients preoperatively.

## 5. Conclusion

Preoperative prediction of ioCSF leakage in patients with PAs during EETS remains challenging, especially for PAs in Hardy Stages A, B, and C. We trained a DNN model using T1CE images which provided a slice-based accuracy of 97.29% on the test dataset, and a patient-based accuracy of 84.61% in the clinical test. This pilot study demonstrates the feasibility of the DNN model in predicting ioCSF leaks in PA patients before surgery.

## Data availability statement

The data analyzed in this study is subject to the following licenses/restrictions: Dataset not available due to ethical restrictions. Requests to access these datasets should be directed to X-JS, shukelson@msn.com.

## Author contributions

X-JS and S-BC: conceptualization and supervision. KZ, JQ, and X-JJ: data curation. HC, KZ, and X-JS: formal analysis. HC and KZ: investigation. HC, W-GC, Z-CX, B-YL, and CL: methodology. X-JS, KZ, JQ, and X-JJ: resources. HC, S-BC, and Z-CX: validation. HC and X-JS: writing original draft. S-BC: writing review and editing. All authors contributed to the article and approved the submitted version.

## Funding

This work was supported by the National Natural Science Foundation of China (NSFC) under Grant 62102133, the Kaifeng Major Science and Technology Project under Grant 21ZD011, the Ji’An Finance and Science Foundation under Grants 20211–085454, 20222–151746, and 20222–151704.

## Conflict of interest

The authors declare that the research was conducted in the absence of any commercial or financial relationships that could be construed as a potential conflict of interest.

## Publisher’s note

All claims expressed in this article are solely those of the authors and do not necessarily represent those of their affiliated organizations, or those of the publisher, the editors and the reviewers. Any product that may be evaluated in this article, or claim that may be made by its manufacturer, is not guaranteed or endorsed by the publisher.
